# Longitudinal computed tomography body composition changes in patients receiving curative radiotherapy for non-small cell lung cancer

**DOI:** 10.1016/j.phro.2026.101055

**Published:** 2026-08-05

**Authors:** Ying Zhang, Sumeet Hindocha, Arjun K. Ghosh, Miguel Garrett Fernandes, Maria A. Hawkins, Charles-Antoine Collins Fekete

**Affiliations:** a Department of Medical Physics and Biomedical Engineering, University College London, Gower Street, London WC1E 6BT, United Kingdom; b University College London Hospitals NHS Foundation Trust, Radiotherapy Physics, 250 Euston Road, London NW1 2PG, United Kingdom; c Cardio-Oncology Service, Barts Heart Centre, St Bartholomew's Hospital, West Smithfield, London EC1A 7BE, United Kingdom; d Cardio-Oncology Centre of Excellence, Hatter Cardiovascular Institute, University College London Hospitals NHS Foundation Trust, 250 Euston Road, London NW1 2PG, United Kingdom; e William Harvey Research Institute, Queen Mary University of London, Charter House Square, Barbican, London EC1M 6BQ, United Kingdom

**Keywords:** NSCLC, Body composition, Cardiac remodelling, Longitudinal changes, Overall survival

## Abstract

**Background and purpose:**

To determine whether longitudinal changes on routine thoracic computed tomography (CT) predict overall survival (OS) in non-small cell lung cancer (NSCLC) after curative radiotherapy and identify dose predictors of adverse tissue changes.

**Materials and methods:**

We performed a retrospective, single-centre study of 231 stage I-IV NSCLC patients who had at least two follow-up scans. In total, 2708 CT scans were analysed (median follow-up, 22 months; range, 1–97). Automated segmentation quantified left ventricular (LV) myocardium, L1 skeletal muscle (SKM) and fat volumes. For each tissue, baseline-normalised trajectories were used to compute monthly rates of change (“velocity”), and non-linear associations with OS were evaluated. Logistic regression identified dose metrics associated with adverse tissue change.

**Results:**

SKM velocity stratified OS (C-index, 0.70): SKM loss < −0.4%/month vs ≥ −0.4%/month, HR 4.41 (95% CI 2.46–7.91, *p* < 0.005). LV-myocardial mass velocity showed a U-shaped relationship with OS (C-index, 0.75): atrophy < −0.3%/month vs stable, HR 4.12 (95% CI 1.65–10.27, *p* < 0.005); hypertrophy >0.3%/month vs stable, HR 7.90 (95% CI 2.82–22.15, p < 0.005). Dose predictors included oesophagus V_10Gy_ for SKM loss (OR, 1.40; *p* = 0.03), Aorta V_5Gy_ for myocardial atrophy (OR, 1.71; *p* = 0.02), and right-atrium V_10Gy_ for myocardial hypertrophy (OR, 1.63; p = 0.02).

**Conclusion:**

Longitudinal CT biomarkers, particularly SKM loss rate and deviation in LV-myocardial mass, are associated with OS after curative NSCLC irradiation. These findings require validation in multicentre studies with more complete clinical information.

## Highlights

• Monthly muscle loss faster than 0.4% separated survival groups (*P* < 0.005).
• Myocardial mass velocity showed a U-shaped relationship with overall survival.
• Oesophagus V_10_ predicted skeletal-muscle loss (odds ratio 1.40; *P* = 0.03).
• Right-atrial V_10_ and fraction dose predicted myocardium hypertrophy.
• Aorta V_5_ (odds ratio 1.71, *p* = 0.02) predicted myocardium atrophy.

## Graphical abstract

**Figure:**
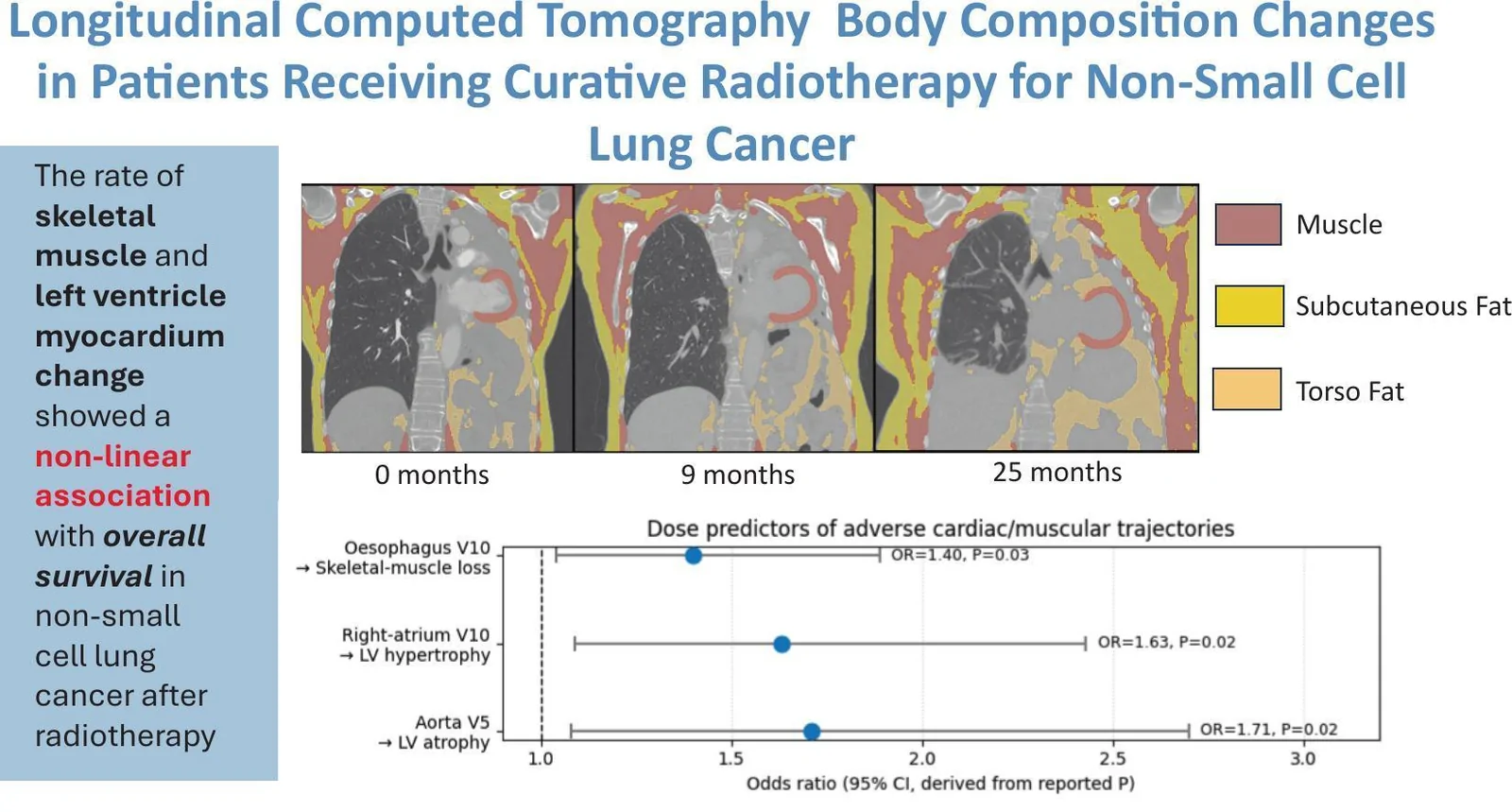

## 1 Introduction

In non-small cell lung cancer (NSCLC), body composition metrics, such as skeletal muscle (SKM) volume, adipose tissue, and aortic calcification, were reported to be associated with baseline physiological resilience, survival and treatment-related side-effects [1], [2], [3], [4]. These non-invasive biomarkers may help identify patients who were more vulnerable, support pre-treatment optimisation, guide supportive care and follow-up.

Body composition is often approximated using body weight or body mass index, but these measures did not distinguish muscle from fat. Computed tomography (CT)-based body-composition analysis provided a more accurate assessment by directly quantifying tissue compartments [5]. This is particularly relevant in patients with NSCLC receiving radiotherapy, where skeletal muscle loss had been shown to predict worse overall survival (OS) [2], [6]. Automated AI methods enable fast, reproducible, population-scale assessments without time-consuming manual segmentation [7].

Beyond muscle and fat, other CT-derived metrics may carry prognostic value. A recent study suggested that a significant reduction in left ventricular (LV) mass is associated with poorer functional status and increased all-cause mortality in advanced cancer patients [8]. Only a few studies have used routine non-electrocardiogram (ECG)-gated CT scans for survival analysis [9], [10]. A high cohort-level correlation between LV mass measured on gated and ungated scans has been reported (*r* = 0.95) [9], but individual-level agreement and potential systematic bias remained uncertain.

Most existing studies either associated radiation dose metrics with anatomical changes [11] or associated those changes with patient survival, typically using only two time points [12], [13]. This approach limits characterisation of longitudinal anatomical patterns across multiple time points.

We hypothesised that longitudinal changes in body composition, including skeletal muscle (SKM) and adipose tissue, and in the irradiated heart over months to years would be associated with overall survival in patients with NSCLC undergoing radical radiotherapy. This study extends previous research by evaluating body composition changes over a longer follow-up period and by including an exploratory assessment of left ventricular (LV) mass using ungated CT. In addition, we investigated treatment-related and dose–volume predictors of adverse longitudinal changes, including severe skeletal muscle loss, LV myocardial atrophy, and LV myocardial hypertrophy.

## 2 Materials and methods

### 2.1 Patient data

We retrospectively identified 451 consecutive patients with stage I–IV non-small cell lung cancer (NSCLC) treated with radical-intent radiotherapy at University College London Hospital between 2015 and 2023. The study received ethics approval in March 2022 and was registered in the UK Integrated Research Application System under project ID IRAS 303606. Of these, 231 patients (51%) had a pre-treatment CT and two post-treatment follow-up CTs available, yielding 2708 CT scans. Median follow-up was 22 months (range, 1–97). Follow-up CT was generally performed every 3 months during the first year and every 6 months thereafter.

Patients were treated with either stereotactic body radiotherapy (SBRT; 34 Gy in 1 fraction to 60 Gy in 8 fractions; *n* = 132) or conventionally fractionated definitive radiotherapy (dRT; 55 Gy in 20 fractions to 66 Gy in 33 fractions, with some patients receiving 30 Gy in 10 fractions; *n* = 99). Both contrast-enhanced and non-contrast scans were included. Images were reconstructed using 55 kernels, grouped as SOFT or SHARP. None were ECG- or respiratory-gated. Clinical variables included age, sex, overall tumour stage, gross tumour volume, treatment type, survival time, and survival status.

Patients could contribute to one or both analysis-specific sub-cohorts, depending on anatomical coverage and scan availability. We aimed to include at least 150 patients in each sub-cohort.

For LV myocardial analysis, we included patients with a pre-treatment CT and at least three follow-up CT scans covering the entire thorax. Multiple time points were required to reduce variability caused by cardiac and respiratory motion on ungated CT. This sub-cohort included 150 patients, each with 3–12 follow-up scans, giving 1665 scans.

For SKM and fat assessment, measurements were obtained at L1 because L3 was not consistently covered on thoracic CT. Although L3 is more commonly used, L1 is a practical alternative when abdominal coverage is limited and correlates strongly with L3-derived measures [14]. Included patients had a pre-treatment CT scan and at least two follow-up CT scans that fully captured the L1 vertebral body and transverse processes. This sub-cohort included 184 patients, each with 2–11 follow-up scans, giving 1647 scans.

### 2.2 Segmentation

Automated 3D segmentations were obtained using TotalSegmentator (Version 2) [7]. Structures included LV myocardium, cardiac chambers, aorta, oesophagus, pulmonary artery, L1 vertebra (body and bridge), SKM, subcutaneous fat, and torso fat. Two board-certified clinical oncologists reviewed the auto-segmented contours for a subset of 30 patients to verify their conformity with accepted clinical standards. The resulting volumes were used for longitudinal analysis. Examples are provided in Supplementary Material A.

### 2.3 Measurement of longitudinal change

To reduce the influence of scan-to-scan variability in ungated imaging on the estimated longitudinal velocity, patient-specific trends were derived from all available scans rather than a single scan pair, providing a more robust estimate of change over time. For each patient, measurements were normalised to the pre-treatment value and fitted against time in months using linear regression. The slope represented the monthly rate of change. The linear assumption is evaluated and supported in Supplementary Material B.

For LV myocardial mass, the rate of change was denoted $v_{\mathrm{myo}}$. Because L1 coverage varied between scans, SKM, subcutaneous fat, and torso fat volumes were divided by the measured L1 length in centimetres before baseline normalisation. The corresponding linear slopes were denoted $v_{\mathrm{skm}}$, $v_{\mathrm{subF}}$, and $v_{\mathrm{torsoF}}$, respectively. Examples of the fitted longitudinal changes are provided in Supplementary Material C. The effects of reconstruction kernel and contrast-agent use on the estimated velocities are reported in Supplementary Material D.

### 2.4 Survival analysis

We examined the associations between longitudinal tissue-change velocities and overall survival using weighted multivariable Cox proportional hazards models implemented in *lifelines* [15]. The LV model included $v_{\mathrm{myo}}$, while the body-composition model included $v_{\mathrm{subF}}$, $v_{\mathrm{torsoF}}$, and $v_{\mathrm{muscle}}$. Prior evidence suggests that organ-volume-change effects on mortality are non-linear e.g. U-shaped for heart, and that departures from normal cardiac size, towards either hypertrophy or atrophy, increased mortality risk [8], [16]. Each velocity was therefore represented using B-spline basis functions [17]. Models were adjusted for age, sex, treatment type (SBRT vs dRT), and gross tumour volume (see Table S3 and Table S5). Overall stage was not included due to high correlation with treatment type(*r* = −0.68). Inverse-probability-of-treatment weights (IPTW) was used to reduce baseline imbalances between SBRT and dRT.

Model stability and predictive performance were assessed using 1000 bootstrap samples. In each sample, the weighted spline Cox model was refitted, and predictors with Wald (*p* < 0.05) were recorded. Predictors selected in more than 60% of samples were retained in the final model. The optimism-corrected concordance index was calculated by subtracting the mean bootstrap optimism from the apparent C-index.

For risk stratification, the fitted spline curves were used to identify values at which the estimated hazard ratio exceeded 1.0 in each bootstrap. The median bootstrap-derived values were used as the final cut-offs defining high-risk and low-risk/stable groups. Hazard ratios for the resulting cardiac and skeletal-muscle risk categories were reported using the stable group as the reference.

### 2.5 Dose deposited in organs related to longitudinal changes

Biological effective dose (BED) was calculated as.

$$\mathrm{BED}=d\left(1+\frac{d}{\alpha /\beta },\alpha /\beta =3\right)$$

where n is the number of fractions, d is the dose per fraction, and α/βwas set to 3 Gy. BED-based dose-volume metrics from V_5Gy_ to V_65Gy_, in 5 Gy increments, were extracted for four heart chambers, left ventricular (LV) myocardium, body, aorta, oesophagus, and pulmonary artery. V_x_ represents the volume of the structure receiving at least x Gy BED.

For dose–response analyses, each biomarker was binarized using the final bootstrap-derived cut-off from the spline Cox analysis. The high-risk group was coded as 1, and the low risk/stable group was coded as 0. Collinearity among dose variables was first reduced by hierarchical clustering of the pairwise correlation matrix into four clusters. Age and total dose per fraction were retained as prespecified covariates. For each outcome model, feature selection was then performed using bootstrap stability selection. In each bootstrap sample, one representative predictor was selected from each feature cluster based on clinical relevance and its association with the outcome, evaluated using Firth logistic regression with Bonferroni correction. VIF-based filtering was then applied to reduce residual multicollinearity (VIF ≤ 3). The 5 predictors with the highest bootstrap selection frequencies were retained, underwent a final VIF check, and were further evaluated using L1-penalized logistic regression with balanced class weights and 5-fold cross-validation [18]. Predictors with non-zero coefficients were included in the final weighted multivariable logistic regression model. Model performance was reported as internally assessed apparent performance. The analysis code is available at https://github.com/YingClara92/BodyComposition.git.

## 3 Results

**Fig. 1:**
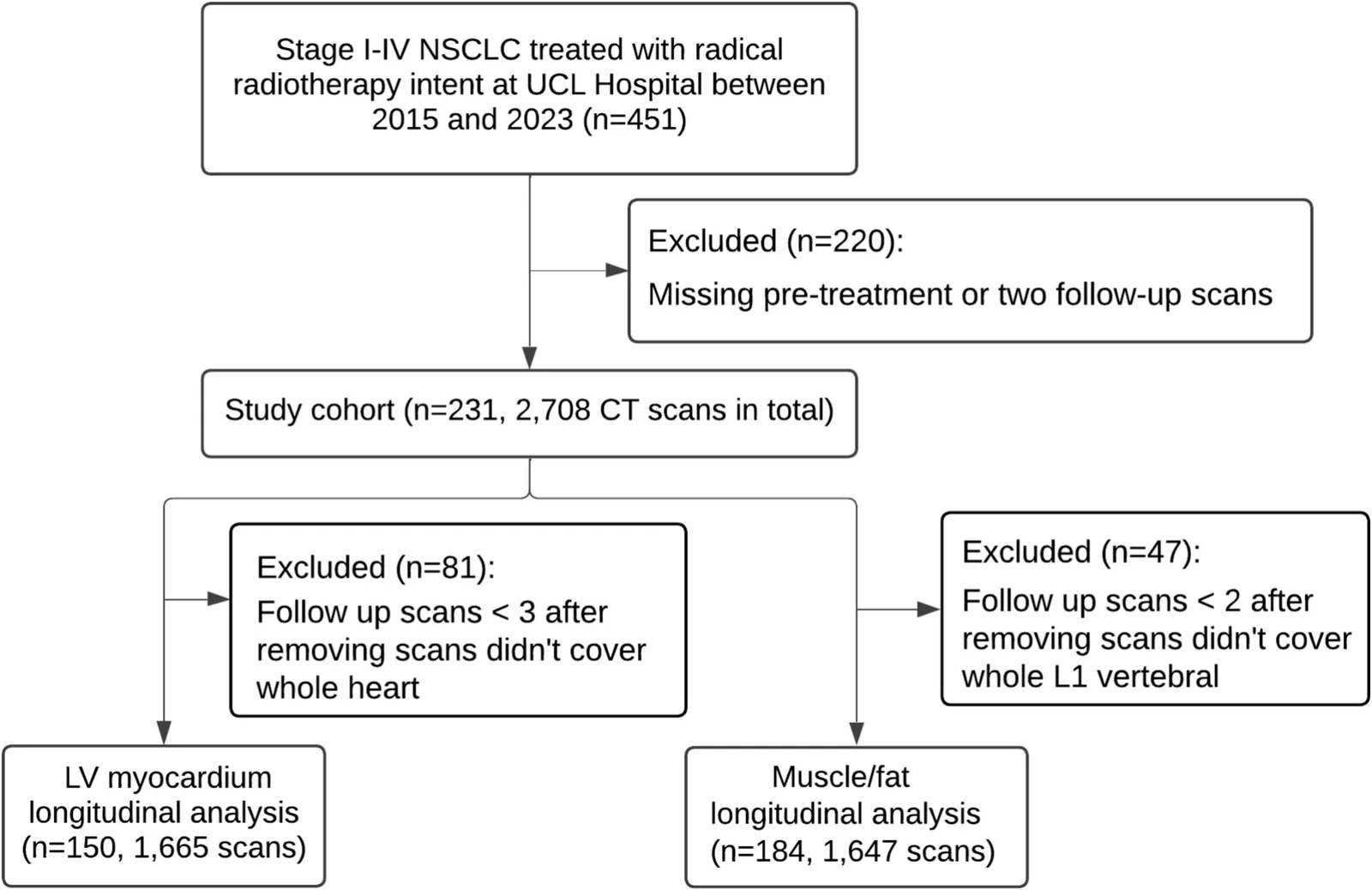
Patient inclusion and exclusion flow chart. NSCLC = non-small cell lung cancer, LV = left ventricular.

Of the 451 patients with stage I–IV NSCLC treated at UCL Hospital between 2015 and 2023, 220 were excluded because pre-treatment or at least two follow-up CT scans were unavailable, leaving 231 patients for analysis (Table 1; Fig. 1).

**Table 1:** Summary of the major clinical characteristics of the patients. The number presents the mean ± standard deviation (SD). SBRT: stereotactic body radiotherapy. dRT: definitive radical radiotherapy. Kolmogorov–Smirnov test was used for p value calculation. Imaging time distribution of dRT and SBRT is shown in Supplementary material A.

	All	dRT (99)	SBRT (132)	p value between SBRT and dRT
**Age [mean ± SD]**	75.0 ± 9.8	70.6 ± 9.2	78.1 ± 9.0	<0.005
**GTV volumes (cm**^**3**^**) [mean ± SD]**	161.6 ± 222.6	329.8 ± 262.0	48.4 ± 71.2	<0.005

**Gender**				
Female	123(53.2%)	56 (56.6%)	67 (50.8%)	0.46
Male	108(46.8%)	43 (43.4%)	65 (49.2%)	

**Overall Stage**				
I	122(52.8%)	8 (8.1%)	114 (86.3%)	<0.005
II	33(14.3%)	28 (28.3%)	5 (3.8%)	
III	60(26.0%)	55 (55.6%)	5 (3.8%)	
IV	7(3.0%)	4 (4.0%)	3 (2.3%)	
Unknown	9(3.9%)	4 (4.0%)	5 (3.8%)	

**RT (Total Dose/Fractions)**				
60- 66Gy/30–33	58 (25.1%)	58 (58.6%)		/
50–55/20	35(15.2%)	35(35.4%)		/
60/15	2 (0.9%)	2 (2.0%)		/
30/10	4 (1.7%)	4 (4.0%)		/
60/8	31 (13.4%)		31 (23.5%)	/
45–55/5	59 (25.5%)		59 (44.7%)	/
30–54/3	38 (16.5%)		38 (28.8%)	/
34/1	4 (1.7%)		4 (3%)	/
**Number of sequential imaging [median ± 95%CI]**	7 [3−13]	8 [3−12]	7 [3–13]	0.38
**Imaging time** **[median ± 95%CI]**	18[1–66]	19[1–69]	1[0–61]	<0.005

After IPTW adjustment, tissue-change velocities differed between SBRT and dRT for subcutaneous fat, torso fat, and LV myocardium, but not for skeletal muscle; however, overall survival did not differ significantly by treatment type in either analysis-specific cohort (Supplementary material E).

We fitted a univariate IPTW Cox proportional hazards model using B-splines with $v_{\mathrm{skm}}$ only because $v_{\mathrm{subF}}$, $v_{\mathrm{torsoF}}$, age, sex, treatment group (SBRT vs dRT), and gross tumour volume did not meet the predefined bootstrap stability criterion for inclusion in the final model. The optimism-corrected C-index was 0.70. Spline modelling improved the concordance index by approximately 7% compared with a linear model (Supplementary Material F). Additional sensitivity analyses using different cropped longitudinal follow-up windows showed that concordance was highest when more complete longitudinal data were used, supporting the added value of incorporating additional longitudinal measurements; details were provided in Supplementary material G.

**Fig. 2:**
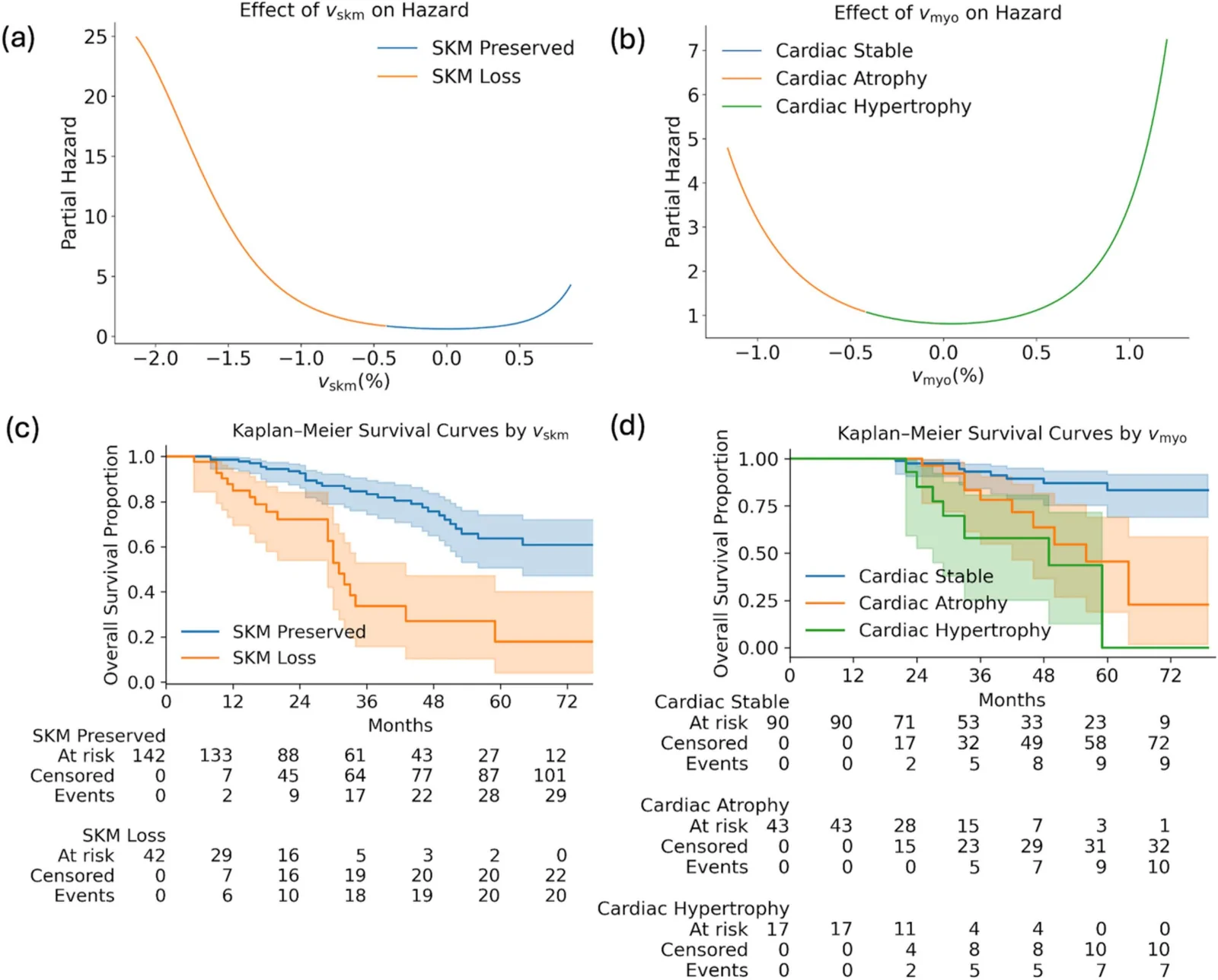
Partial hazard plot-based Kaplan-Meier (KM) survival analysis. (a) is the partial hazard plot $v_{\text{muscle}}$. SKM loss ($v_{\text{muscle}}<-0.4$) represents patients with significant skeletal muscle loss and SKM preserved ($v_{\text{muscle}}\geq -0.4$) are those without. (b) is the KM survival curve of groups for $v_{\text{muscle}}$. (c) is the partial hazard plot $v_{\mathrm{myo}}$. Cardiac stable is the LV myocardial mass stable group ($-0.3\leq v_{\mathrm{myo}}\leq 0.3$), Cardiac atrophy ($v_{\mathrm{myo}}\leq -0.3$) is the LV myocardial mass loss group, and Cardiac hypertrophy ($v_{\mathrm{myo}}>0.3$) is the LV myocardial mass gain group. (d) is the KM survival curve of groups for $v_{\mathrm{myo}}$.

The spline-based analysis identified −0.4±0.1%/month as the cut-off value for $v_{\mathrm{skm}}$ across bootstrap resamples. The spline-based partial hazard plot from all data (in Fig. 2a) declined monotonically and then nearly plateaued, with only a modest upturn. We defined two groups using a median bootstrap-derived threshold: 1) SKM preserved/minor loss, including patients with increased, stable SKM, or minor SKM loss ($v_{\mathrm{skm}}\geq -0.4\%/\mathrm{month}$), where the hazard was lower and stable; and 2) Severe SKM loss, including patients with significant SKM loss ($v_{\mathrm{skm}}<-0.4\%/\mathrm{month}$), which was associated with a sharp increase in hazard. Patients with SKM loss had a substantially higher risk of death than those with preserved SKM (HR 4.41, 95% CI 2.46–7.91, *p* < 0.005; Table 2). The corresponding KM curves differed significantly between the two groups (Fig. 2b).

**Table 2:** Hazard ratios for overall survival by cardiac and skeletal muscle categories, using the stable group as the reference.

Category	HR (vs stable)	95% CI	p value
Cardiac Hypertrophy	7.90	2.82–22.15	<0.005
Cardiac Atrophy	4.12	1.65–10.27	<0.005
SKM Loss	4.41	2.46–7.91	<0.005

We also fitted a univariate spline Cox model for $v_{\mathrm{myo}}$ in the LV myocardium cohort because age, sex, treatment group (SBRT vs dRT), or gross tumour volume did not meet the predefined bootstrap stability criterion for inclusion in the final model. The spline model captured a U-shape hazard curve and improved predictive accuracy with optimism-corrected C-index of 0.75 (+22% vs. linear). A standard linear Cox model failed to detect any association between $v_{\mathrm{myo}}$ and OS (Supplementary material F), likely because the U-shaped hazard masked the effect, with opposing slopes that average to ∼0. The bootstrapping identified the thresholds at which HR > 1, which were − 0.3%/month (95% CI: −0.4 to −0.2) and 0.3%/month (95% CI: 0.2 to 0.5), respectively. The partial hazard curve in Fig. 2c was asymmetric and U-shaped: the hazard was lowest near stable mass, rose steeply for hypertrophy (positive $v_{\mathrm{myo}}$) and rose more gradually for atrophy (negative $v_{\mathrm{myo}}$).

We defined three categories: 1) cardiac stable: $-0.3\%/\mathrm{month}\leq v_{\mathrm{myo}}\leq$0.3%/month, 2) cardiac atrophy: $v_{\mathrm{myo}}<$ -0.3%/month, and 3) cardiac hypertrophy: $v_{\mathrm{myo}}>$0.3%/month. Compared with patients in the stable group, both cardiac hypertrophy (HR 7.9, 95% CI 2.82–22.15, *p* < 0.005) and cardiac atrophy (HR 4.12, 95% CI 1.65–10.27, p < 0.005) were associated with increased mortality (Table 2). KM analysis showed significantly poorer survival in both the atrophy and hypertrophy groups compared with the stable group (Fig. 2d). Although the log-rank comparison between the cardiac atrophy and hypertrophy groups did not reach statistical significance (*p* = 0.12), the KM estimates at 30 months post-treatment suggested a clinically meaningful divergence in survival probabilities (92% [95% CI: 72–98%] vs. 70% [95% CI, 38–87%]).

Higher low-dose exposure to selected thoracic organs was associated with adverse longitudinal changes in SKM and LV myocardial mass (Table 3). Oesophageal V_10Gy_ was independently predictive of SKM loss (OR 1.40, 95% CI 1.04–1.89; *p* = 0.03), albeit with modest discrimination (AUC 0.63, 95% CI: 0.53–0.73). For LV myocardial atrophy, aorta V_5Gy_ was a significant predictor (OR 1.71, 95% CI 1.08–2.70; *p* = 0.02), while heart myocardium V_10Gy_ showed a weaker association that did not reach statistical significance (*p* = 0.09). The LV myocardial atrophy model showed discrimination of 0.71 (95% CI: 0.61–0.81). Heart right atrium V_10Gy_ (OR 1.63, 95% CI 1.09–2.43; p = 0.02) and total dose fraction (OR 1.51, 95% CI 1.02–2.22, *p* = 0.04) were significantly associated with LV myocardial hypertrophy and demonstrated AUC of 0.65 (95% CI: 0.51–0.78).

**Table 3:** Logistic regression results. *p*-values are two-sided.

Outcome	Predictor range	Odds ratio (95% CI)	p	AUC (95% CI)	Accuracy (95% CI)
Skeletal muscle loss	Oesophagus V_10Gy_ (cm^3, mean ± SD) 12.7 ± 12.6	1.40 (1.04–1.89)	0.03	0.63 (0.53–0.73)	0.69 (0.54–0.71
	Heart atrium right V_20Gy_ (cm^3, mean ± SD) 6.8 ± 17.5	1.32 (0.98–1.79	0.07		
	Age (years, mean ± SD) 75.8 ± 8.8	1.11 (0.82–1.49)	0.50		
Cardiac Atrophy	LV myocardium V_10Gy_ (cm^3, mean ± SD) 12.5 ± 25.5	1.50 (0.94–2.39)	0.09	0.71 (0.61–0.81)	0.71 (0.61–0.77)
	Aorta V_5Gy_ (cm^3, mean ± SD) 111.2 ± 99.2	1.71 (1.08–2.70)	0.02		
	Age (years, mean ± SD) 75.8 ± 8.8	1.37 (0.93–2.04)	0.11		
	TotalDosePerFraction	1.17 (0.79–1.74)	0.42		
Cardiac Hypertrophy	Heart atrium right V_10Gy_ (cm^3, mean ± SD) 9.4 ± 18.7	1.63 (1.09–2.43)	0.02	0.65 (0.51–0.78)	0.65 (0.45–0.71
	TotalDosePerFraction (Gy, middle [95% CI]) 7.5 [2–34]	1.51 (1.02–2.22)	0.04		

Footnote: OR = odds ratio; CI = confidence interval. Dose reported is biological effective dose.

AUC = area under the receiver operating characteristic (ROC) curve, presented with 95% CI.

Accuracy = proportion of correctly classified patients using a predicted probability cut-off of 0.5.

## 4 Discussion

This study showed that longitudinal CT-derived biomarkers provided prognostic information in NSCLC patients treated with curative-intent radiotherapy. Severe skeletal muscle loss and LV myocardial remodelling, including both atrophy and hypertrophy, were associated with poorer survival. Low-dose thoracic dose-volume metrics were also associated with these adverse changes, suggesting potential nutritional and cardiopulmonary pathways that are discussed below.

Our results were broadly concordant with, and extend, existing literature. Al-Sawaf et al. [4] (*n* = 188) reported that >20% of visceral fat or > 10% of SKM loss between diagnosis and relapse was associated with the poorer OS in NSCLC patients. Chaunzwa et al. [19] (*n* = 1791) measured muscle and fat changes before treatment and 4–8 weeks after in metastatic NSCLC patients. They observed that SKM loss, but not fat loss, correlated with survival outcomes. Similarly, we found that SKM loss predicted worse OS and further showed a non-linear SKM muscle-loss–mortality relationship. Oesophageal V_10Gy_ was associated with skeletal muscle loss, which may reflect an indirect nutritional pathway rather than a direct radiobiological effect on skeletal muscle. Low-dose oesophageal irradiation may contribute to oesophagitis, reduced intake, and subsequent muscle loss, supporting the hypothesis that treatment-related side effects may partly drive post-treatment body-composition changes [20].

Although L3 is the standard level for CT-based body composition assessment, we used L1 because routine thoracic follow-up CT scans did not consistently include L3 [21]. L1 has been used as a pragmatic alternative in lung-cancer cohorts for survival analyses [22], [23], [24]. Respiratory-phase differences may have introduced variability in automated L1 segmentation because dedicated respiratory-motion correction was not applied. However, estimating baseline-normalised velocity across multiple time points may reduce random inter-scan variability. Therefore, our findings should be interpreted as L1-derived longitudinal body-composition biomarkers.

For LV myocardial mass loss, aorta V_5Gy_ was significant, whereas heart myocardium V_10Gy_ showed only a non-significant trend (*p* = 0.09). Zhang et al. linked low-dose LV myocardial exposure, particularly V_10Gy_, to declines in left ventricular ejection fraction [25]. However, the biological plausibility of low-dose exposure to the aorta or myocardium causing ventricular atrophy remains unclear. These associations may reflect indirect radiation effects, vascular injury, systemic illness, or residual confounding, and should therefore be interpreted cautiously [26].

In the cardiac-hypertrophy subgroup, higher dose per fraction and increased right atrium V_10Gy_ were associated with LV myocardial hypertrophy. This finding may be relevant because the cohort included SBRT patients treated with different fractionation schemes. These findings are consistent with preclinical data showing that a single high dose (up to 8Gy) can induce significant myocardial fibrosis in animal models, whereas the same total dose delivered in smaller fractions appears less harmful to cardiomyocytes [27]. However, this result should be interpreted cautiously because the cardiac-hypertrophy subgroup was small and the confidence intervals were wide. Two representative cases in Supplementary material H showed reduced left lung volume with cardiac enlargement or displacement after treatment. Therefore, right atrium V_10Gy_ may reflect broader cardiopulmonary irradiation rather than directly causing LV myocardial hypertrophy. Some changes may also reflect cardiac motion or segmentation uncertainty because the CT scans were not ECG-gated.

Methodologically, patient-specific linear slopes and cubic spline Cox models accounted for variable scan timing and potential non-linear associations with survival. Routine ungated CT images increased clinical relevance but introduced measurement error. Patients with body-composition or myocardial change velocities close to the bootstrapped cut-off values should therefore be interpreted with caution, particularly when values fall within the confidence intervals of these thresholds. Additional clinical information, such as ECG findings, blood tests, and echocardiography, may support interpretation. Future validation using ECG-gated CT is required to assess individual-level agreement and the stability of these categories [28], [29], [30].

The prognostic value of the $v_{\mathrm{myo}}$ and$v_{\mathrm{skm}}$ may have several clinical implications. These imaging biomarkers may reflect downstream effects of nutritional status, treatment-related side effects, physical activity, and cardiopulmonary stress. They may help identify patients who could benefit from early supportive interventions, including dietary counselling, exercise programmes, or targeted cardiac surveillance.

This study has several limitations. First, it was a single-centre retrospective analysis, which may limit generalisability. Second, important non-imaging factors were not available, including ECOG performance status, cardiac comorbidities, chemotherapy details, and immunotherapy use. These missing data were potential sources of bias because they may have influenced both survival and longitudinal changes in body composition or cardiac morphology. The relatively high mean age of the cohort may have introduced further bias, as older patients are more likely to have frailty, sarcopenia, cardiovascular disease, and other comorbidities at baseline. Third, follow-up CT scans occurred at variable times, and the requirement for multiple scans may have introduced survivor bias. Fourth, LAD coronary artery dose was not assessed because TotalSegmentator did not provide LAD contours, which limited assessment of cardiac substructure dose effects. Finally, the dose-based models showed only moderate discrimination, with AUC values of approximately 0.6–0.7. Therefore, the reported dose-volume metrics should be regarded as candidate planning parameters for further investigation rather than definitive clinical thresholds. Prospective multi-centre validation with systematic clinical data collection is needed.

This study shows that longitudinal CT-derived biomarkers provide prognostic information in NSCLC patients treated with curative-intent radiotherapy. Skeletal muscle loss and LV myocardial remodelling were associated with poorer survival. These routine CT-based measures may help identify vulnerable patients and generate dose-planning hypotheses, but prospective validation is needed before clinical implementation.

## Work originated institution

Department of Medical Physics and Biomedical Engineering, University College London, Gower Street, London WC1E 6BT, United Kingdom.

## Data sharing statement

The data that support the findings of this study are not publicly available due to ethical restrictions.

## CRediT authorship contribution statement

**Ying Zhang:** Writing – original draft, Visualization, Methodology, Investigation, Formal analysis, Data curation, Conceptualization. **Sumeet Hindocha:** Writing – review & editing, Conceptualization. **Arjun K. Ghosh:** Writing – review & editing. **Miguel Garrett Fernandes:** Writing – review & editing. **Maria A. Hawkins:** Writing – review & editing, Supervision, Funding acquisition. **Charles-Antoine Collins Fekete:** Supervision, Funding acquisition.

## Funding

This project is funded by UKRI Future Leaders Fellowship, No. MR/T040785/1 and the Radiation Research Unit at the Cancer Research UK City of London Centre Award C7893/A28990 and CRUK RRNPSF-Jan21\100001 grant. Maria Hawkins is supported by the National Institute for Health and Care Research University College London Hospitals Biomedical Research Centre.

## Declaration of competing interest

The authors declare that they have no known competing financial interests or personal relationships that could have appeared to influence the work reported in this paper.
