# Supplementary material for: Longitudinal computed tomography body composition changes in patients receiving curative radiotherapy for non-small cell lung cancer
Source: Phys Imaging Radiat Oncol. 2026 Aug 5;40:101055. doi: 10.1016/j.phro.2026.101055 (PMC13499410; doi:10.1016/j.phro.2026.101055)
Supplement: Supplementary file 1 [file mmc1.pdf]

Supplementary A: Examples of AI segmentation.

Examples of AI segmentation is illustrated in Figure S1.

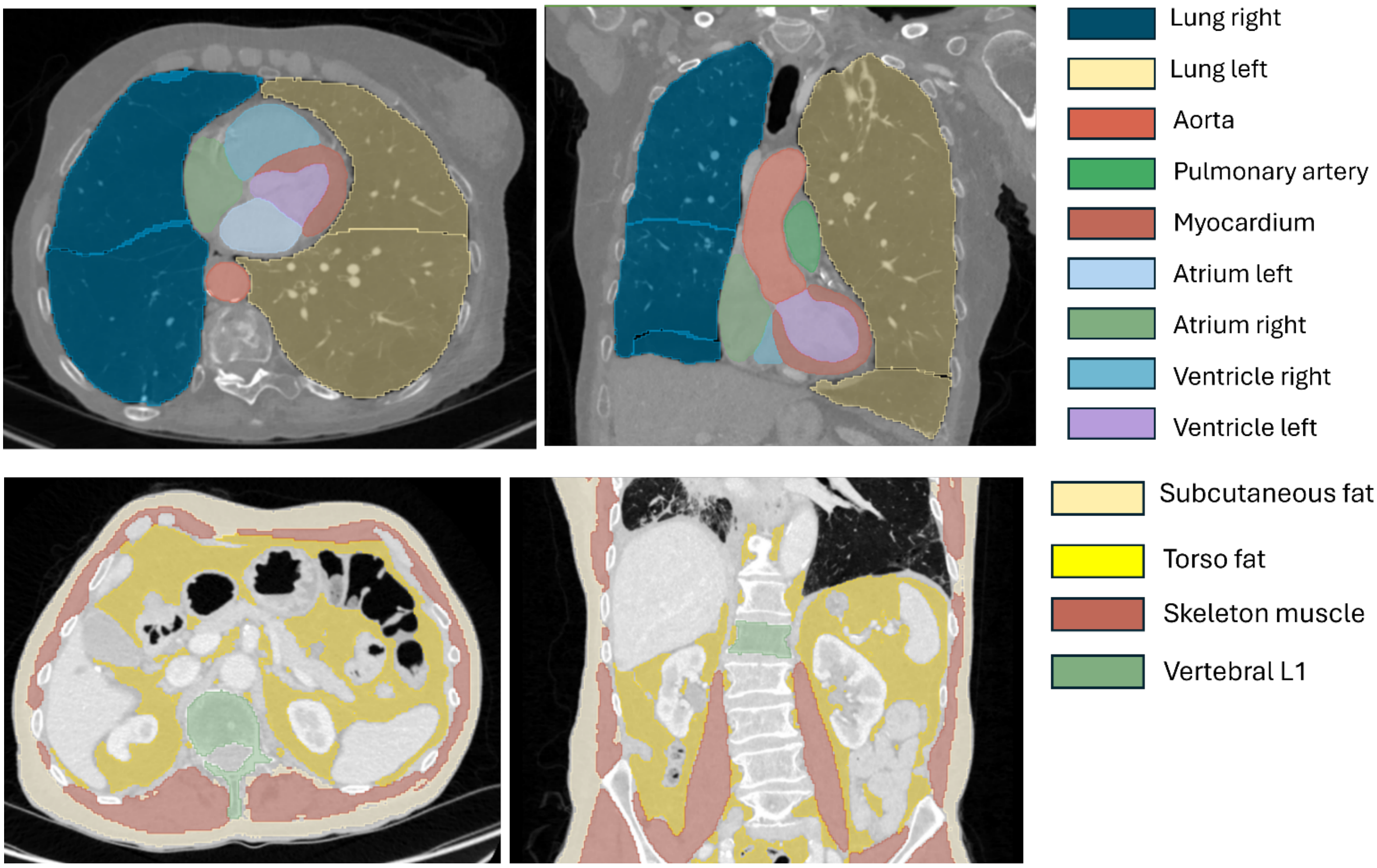

Figure S1. AI segmentation results. (a) presents the AI segmentations of cardiac and pulmonary structures. (b) presents the AI segmentations of muscle and fat and vertebral L1.

The distribution of imaging time is shown in Figure S2.

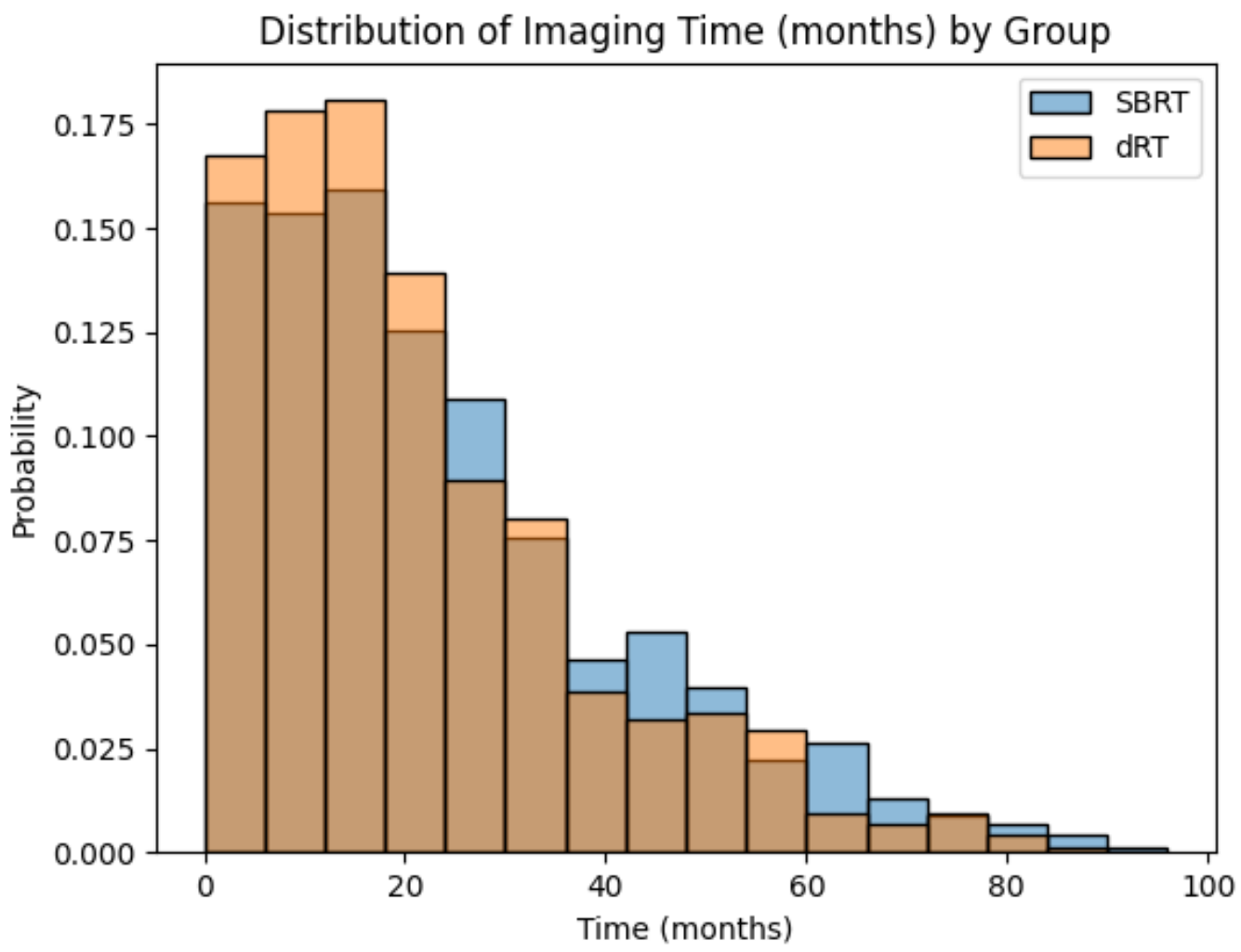

Figure S2 Distribution of Imaging time between SBRT and dRT.

Supplementary B: The validation of linear assumption

To assess whether the assumption of a linear change over time was reasonable, we compared two regression models. The first model included time as a simple linear term. This model assumes that the average volume changes at a constant rate over time. The second model used a natural cubic spline for time with 3 degrees of freedom. This spline model is more flexible and allows the relationship between time and volume to bend or curve.

The linear and spline models were compared using a likelihood ratio test with a significance level of $\alpha = 0.05$. The purpose of this test was to determine whether the more flexible spline model provided a significantly better fit than the simpler linear model. If the test was significant, this would suggest that the linear assumption may not be appropriate. If the test was not significant, this would indicate that there was no strong evidence that a nonlinear time trend improved the model fit.

This comparison was performed separately for each non-stable cohort because some patients showed increasing volume over time, whereas others showed decreasing volume. If these opposite patterns were analysed together, they could cancel each other out and make the overall time trend appear weaker or less clear. Separate analyses were conducted for cardiac hypertrophy, cardiac atrophy, and severe skeletal muscle loss cohort. The likelihood ratio test was not significant for cardiac hypertrophy, cardiac atrophy, or severe skeletal muscle loss cohort, with p-values of 0.40, 0.15, and 0.99, respectively. These results indicate that the spline model did not fit the data significantly better than the linear model. Therefore, there was no strong evidence against the use of a linear time effect in these cohorts.

Supplementary C: The illustration of linear regression for longitudinal data points

Due to cardiac motion, LV myocardium volume measurements showed greater fluctuation across CT scans than skeletal muscle measurements at the L1 vertebral level. Therefore, estimation of v_{myo} may be more sensitive to the number and timing of available longitudinal imaging time points. To illustrate how volume-change velocity was estimated, Figure S3 shows representative examples of linear regression fitting for LV myocardium, skeletal muscle at L1, subcutaneous fat at L1, and torso fat at L1.

For each example, the measured volume values are plotted against time, and a linear regression line is fitted to the longitudinal measurements. The slope of the fitted line represents the estimated volume-change velocity used in the analysis. The left and right panels show two representative patient examples for each tissue type, illustrating differences in longitudinal measurement patterns and fitted velocity estimates.

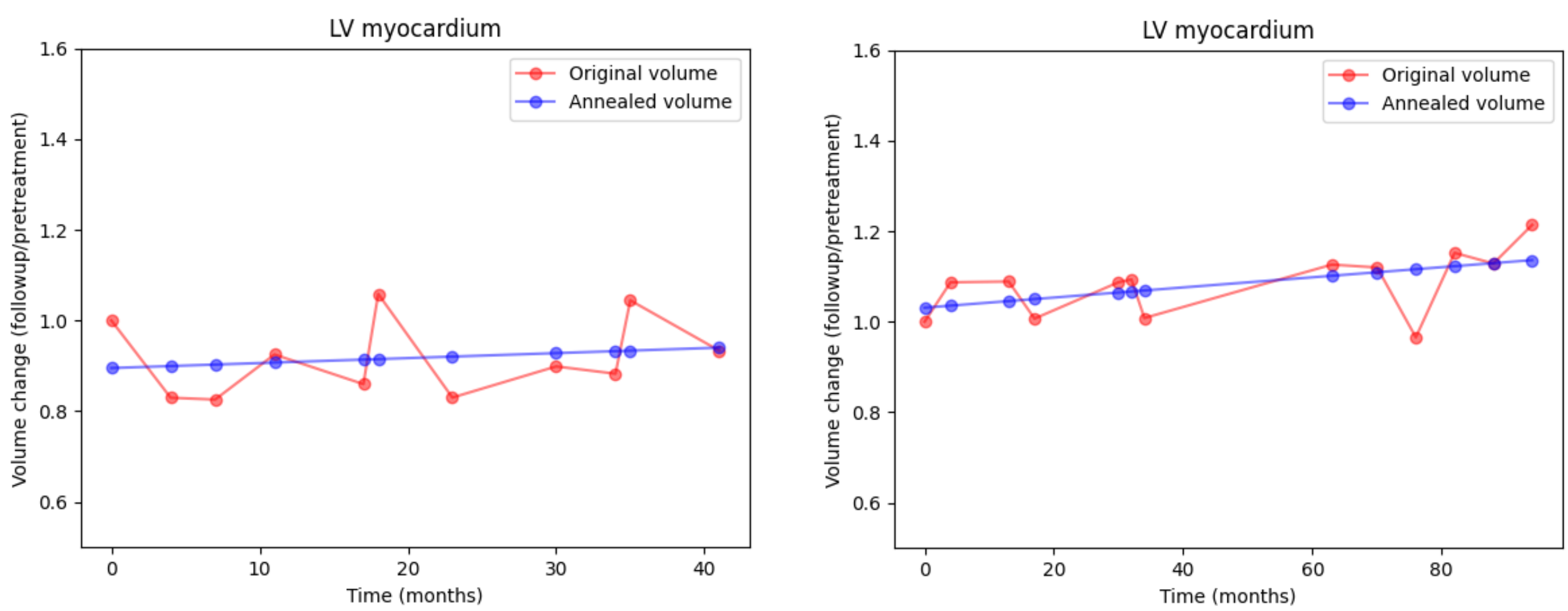

(a) Linear regression fitting for LV myocardium

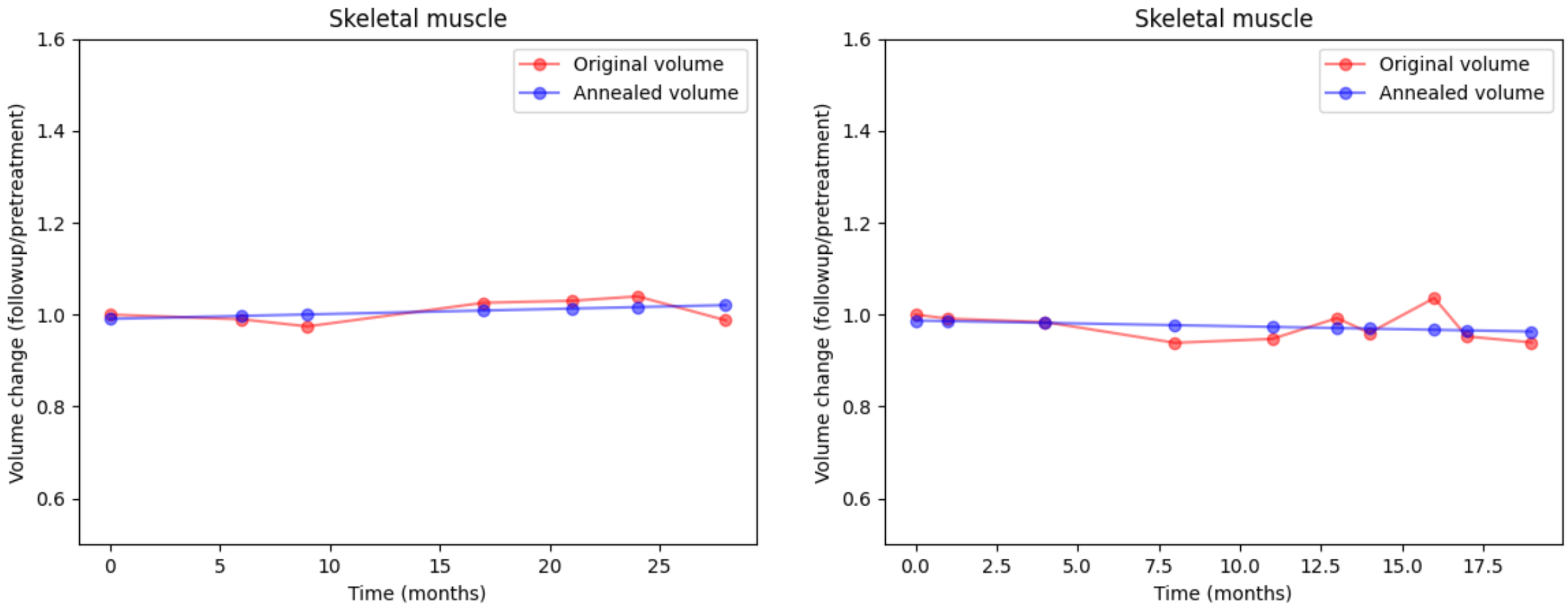

(b) Linear regression fitting for skeletal muscle at vertebral L1

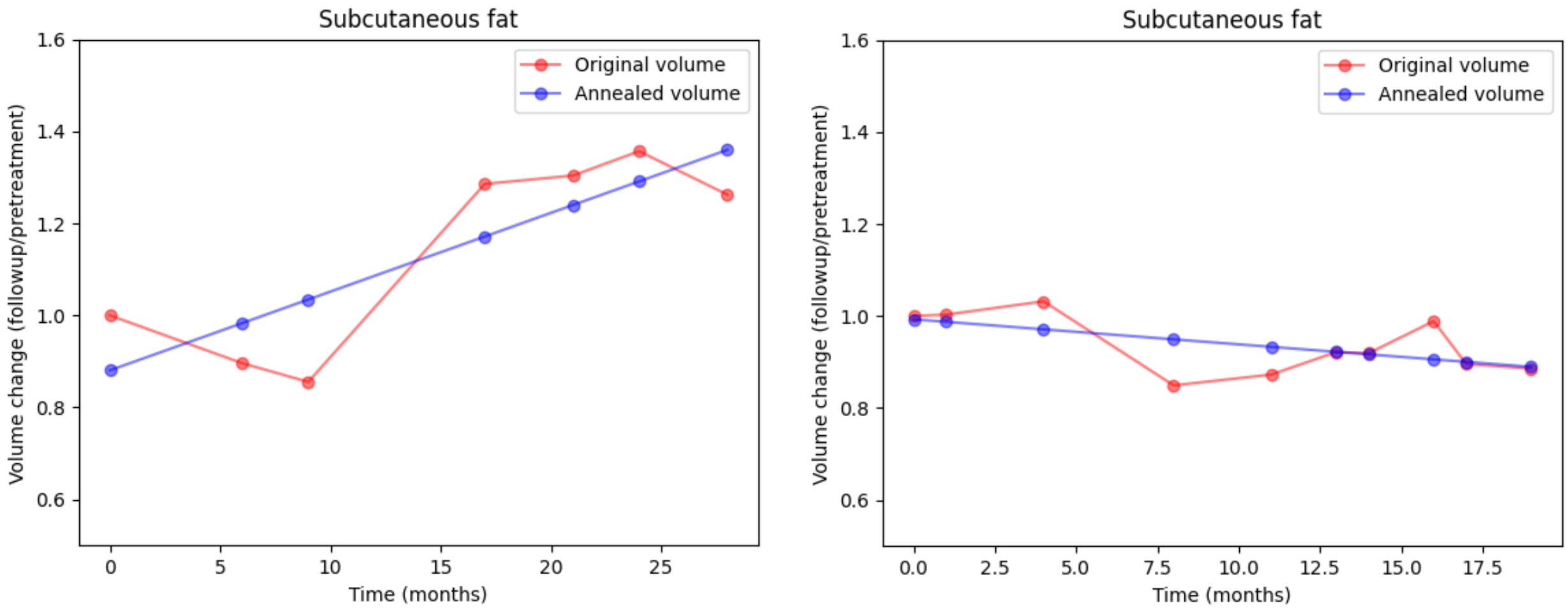

(c) Linear regression fitting for subcutaneous fat at vertebral L1

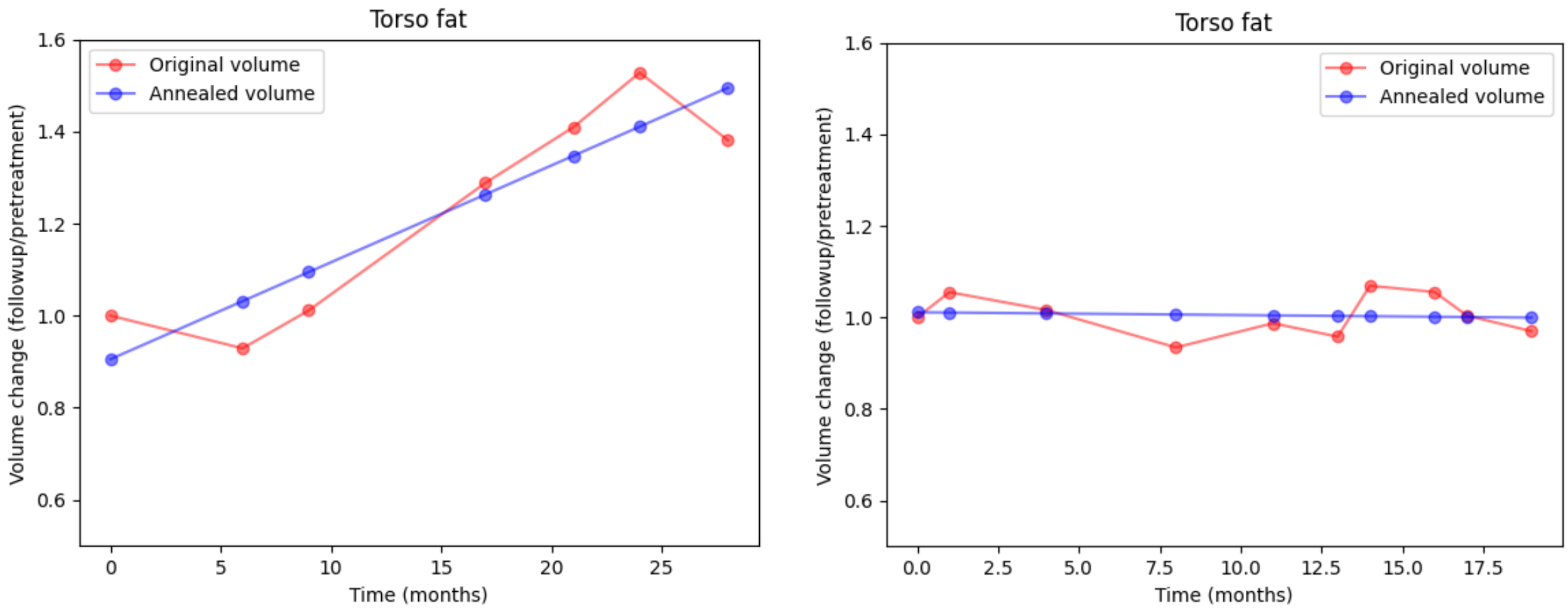

(d) Linear regression fitting for torso fat at vertebral L1

Figure S3. Representative examples of linear regression fitting for longitudinal volume measurements.

Supplementary D The influence of different CT reconstruction kernels on the velocity

In our dataset, some CT scans of the same anatomical region were reconstructed using different kernels and/or acquired with different contrast-agent status. To assess how these image-series differences may influence the longitudinal velocity estimates used in this study, we calculated the variability in segmented volume across available image versions of the same anatomy and expressed it on the same scale as the volume-change velocity:

$$\Delta v_{image} = \frac{V_{max} - V_{min}}{V_{baseline} \times \Delta t} \times 100$$

Where V_{max} and V_{min} represents the maximum and minimum segmented volume measured from CT images of the same anatomical region across different reconstruction kernels and/or contrast-agent status. $V_{baseline}$ is the baseline segmented volume, and $\Delta t$is the time interval from baseline to the last imaging time in months. Δv_{image} represents the potential influence of image-series differences on the estimated volume-change velocity and is reported in %/month.

The results are shown in Table S1. The mean image-series-related variability was low for both myocardium and skeletal muscle, with average values of 0.06%/month for myocardium and 0.12%/month for skeletal muscle.

Table S1. The summary of Δv_{image} for LV myocardium and skeletal muscle.

LV Myocardium (n=547 pair)		Skeletal Muscle (n=292 pair)	
mean	0.06	mean	0.12
std	0.07	std	0.13
min	1e-5	min	1e-5
25%	0.01	25%	0.03
50%	0.04	50%	0.08
75%	0.07	75%	0.16
max	0.8	max	0.77

Supplementary E Baseline (pre-treatment) values for each tissue type

Kaplan–Meier (KM) survival curves show that neither the 150-patient LV myocardium cohort nor the 184-patient muscle/fat cohort showed a significant difference in OS between SBRT and dRT groups (both $p > 0.05$; Figure S4).

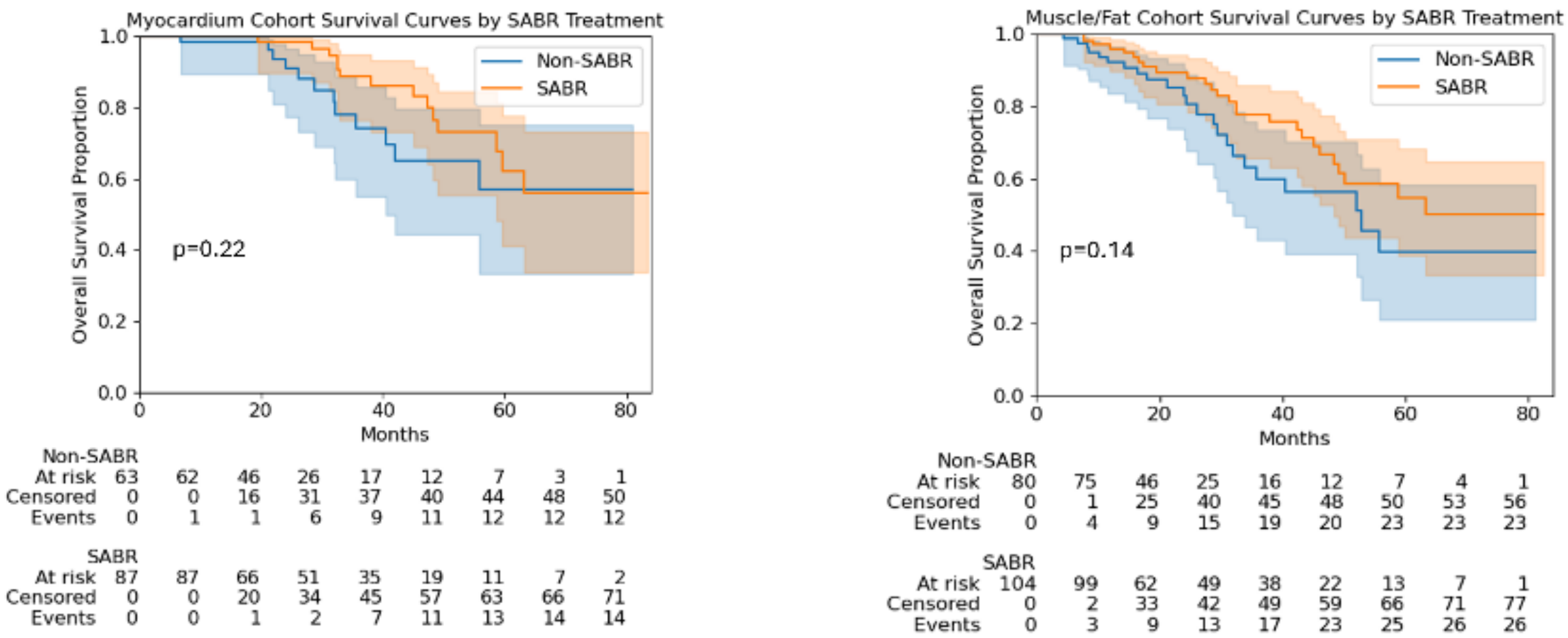

(a) (b)

Figure S4 Kaplan–Meier survival curves stratified by RT type: (a) LV myocardium analysis subgroup; (b) Muscle/fat analysis subgroup.

We summarised baseline (pre-treatment) values for each tissue type in Table S2. There is no significant difference between SBRT and dRT in baseline LV myocardial mass (p=0.8). Compared with dRT, SBRT patients have lower baseline muscle volume (p=0.05).

Table S2 The baseline (pre-treatment) volume of each tissue types. SKM, subcutaneous fat, and torso fat volumes expressed per-cm (divided by measured L1 length) and normalised to baseline. Mann–Whitney U p-values comparing SBRT vs dRT.

Baseline volume(**cm^3**)	dRT	SBRT	P value
LV Myocardium	102.8+29	98.7+23	0.80
Skeletal Muscle	8.9+2.1	8.2+2.1	0.05
Subcutaneous fat	14+11.4	10.4+6.4	0.07
Torso fat	12.4+8.9	10.2+7.6	0.13

To reduce baseline imbalance between the SBRT and dRT groups, inverse-probability-of-treatment weighting (IPTW) was applied. Propensity scores were estimated using a logistic regression model with treatment modality as the dependent variable and baseline age, sex, and GTV volume as predictors. Overall stage was not included because it was highly correlated with treatment modality ($|r| > 0.5$). For each patient, the IPTW weight was calculated as the inverse probability of receiving the treatment actually received. Covariate balance before and after weighting was assessed using absolute standardised mean differences (SMDs), with lower SMD values indicating better balance. After weighting, all included covariates showed substantially reduced SMD values, indicating improved balance between treatment

groups (Table S3). IPTW-weighted linear regression was then used to evaluate the association between treatment modality and longitudinal tissue-change velocities.

Table S3 Absolute Standardised Mean Difference Before and After Weighting

Covariate	Absolute_SMD_Unweighted	Absolute_SMD_Weighted
age	0.69	0.04
GTV volume	1.20	0.09
patient_sex	0.12	0.01

We compared v_{muscle}, v_{subF}, v_{torsoF} and v_{myo} between SBRT and dRT patients (Table S4). In the muscle/fat cohort (58% SBRT), the p value in the multivariable linear regression showed a significant group difference for fat-change velocities ($p<0.05$): SBRT patients lost subcutaneous fat (per-cm volumes) by ~2.6%/year and torso fat ~3%/year, whereas dRT patients gained subcutaneous fat ~26.3%/year and torso fat ~16%/year. In the LV myocardial cohort (56.5% SBRT), LV myocardial mass declined by an additional 1.2%/year in dRT versus SBRT, which is also significant ($p=0.04$). Muscle-loss rates were similar across groups ($p=0.77$).

*Table S4 Comparison of tissue-change velocities (mean ± SD) for LV myocardial mass (v_{myo}), SKM (v_{muscle}), subcutaneous Fat (v_{subF}) and torso Fat (v_{torsoF}) between SBRT and dRT patients. Between-group differences were reported without adjustment and adjustment using multivariable analysis. Values represent the monthly percent volume change (**cm**^{3}/month) from baseline, assuming a linear trend. Please note that the volume of SKM, subcutaneous fat, and torso fat expressed as per-cm (divided by measured L1 length).*

Velocity	*dRT* (%/month)	*SBRT* (%/month)	*Unadjusted difference [*SBRT-dRT] (%/month)	*Adjusted treatment [*SBRT-dRT] (%/month) *effect*	*Adjusted treatment p value*
v_{muscle}	-0.24±0.61	-0.21±0.48	0.03	0.03	0.77
v_{subF}	2.19±9.99	-0.22±2.12	-2.41	-3.14	0.01
v_{torsoF}	1.33±5.36	-0.25±1.88	-1.58	-1.95	0.004
v_{myo}	-0.14±0.37	-0.04±0.36	0.1	0.15	0.04

We fitted the linear Cox model with baseline covariate adjusted for treatment type. The optimism-corrected C-index is 0.49.

Table S5. Multivariable Linear Cox Model with baseline covariate

Variable	HR (95%CI)	p-value
SABR	0.69 (0.28-1.70)	0.42
age	1.06 (0.65-1.72)	0.82
Gender (Female:1, Male:0)	0.69 (0.29-1.64)	0.4
GTV volume	0.68 (0.39-1.21)	0.19

Supplementary F: The standard linear Cox model

Because v_{torsoF} and v_{subF} were highly correlated, we fitted two separate multivariable linear Cox proportional-hazards models, each including v_{muscle} plus one of the fat features in Table S6 and Table S7 respectively.

Table S6. Multivariable Linear Cox Model 1: v_{muscle} and v_{torsoF}

Variable	HR (95%CI)	p-value
$\boldsymbol{v_{muscle}}$	0.25 (-0.81-0.44)	<0.005
$\boldsymbol{v_{torsoF}}$	1.05 (0.14-1.15)	0.35

Table S7. Multivariable Linear Cox Model 2: v_{muscle} and v_{subF}

Variable	HR (95%CI)	p-value
$\boldsymbol{v_{muscle}}$	0.27 (-0.75-0.47)	<0.005
$\boldsymbol{v_{subF}}$	0.99 (0.06-1.07)	0.83

In both models, only v_{muscle} was a significant predictor of overall survival. Neither v_{subF} or v_{subF} had a significant effect.

When we fitted a linear Cox model with v_{muscle} alone in Table S8, the optimism-corrected C-index was 0.63, lower than that of the spline-based Cox model (c-index = 0.76).

Table S8. Univariable Linear Cox Model 3: v_{muscle}

Variable	HR (95%CI)	p-value
$\boldsymbol{v_{muscle}}$	0.28 (0.11-0.73)	0.06

In contrast to the spline-based Cox model, the linear univariable Cox model using v_{myo} yielded a optimism-corrected C-index of 0.53 (see Table S9) and did not detect a significant association with overall survival. This discrepancy likely arises because a U-shaped relationship can produce an almost zero linear coefficient in a standard Cox model, whereas a spline-based approach can uncover its underlying risk pattern.

Table S9. Univariable Linear Cox Model using v_{myo}.

Variable	HR (95%CI)	p-value
$\boldsymbol{v_{myo}}$	0.92 (0.03-3.70)	0.45

Supplementary G: Validation of additional benefits of using longitudinal data

We would like to validate that adding the additional data points would lead to higher concordance index using skeleton muscle at vertebral L1.

Our results reported in the manuscript used all the data points we have for the longitudinal features. In this Supplementary section, we tried to crop the data point by specific time points (20 months, 36 months, 48 months) for survival analysis. Wilcoxon p value between estimated velocity from cropped data points and full data points, and the concordance index using velocity from cropped data points are listed in Table S10.

Table S10. The Wilcoxon p value between estimated velocity from cropped data points and full data point, and concordance index based on the estimated velocity from cropped data points. The number in bracket indicates the patient number included in the Cox model.

Evaluation Metrics	v-20 months	v-36 months	v-48 months
Wilcoxon p value (compared with v_{full})	0.18	0.21	0.14
Concordance index	0.67 (148)	0.65 (165)	0.70 (175)

Then we plotted velocity difference $v_{full} - v_{cropped}$, between the estimated velocity with all data points and with cropped data points in Figure S5.

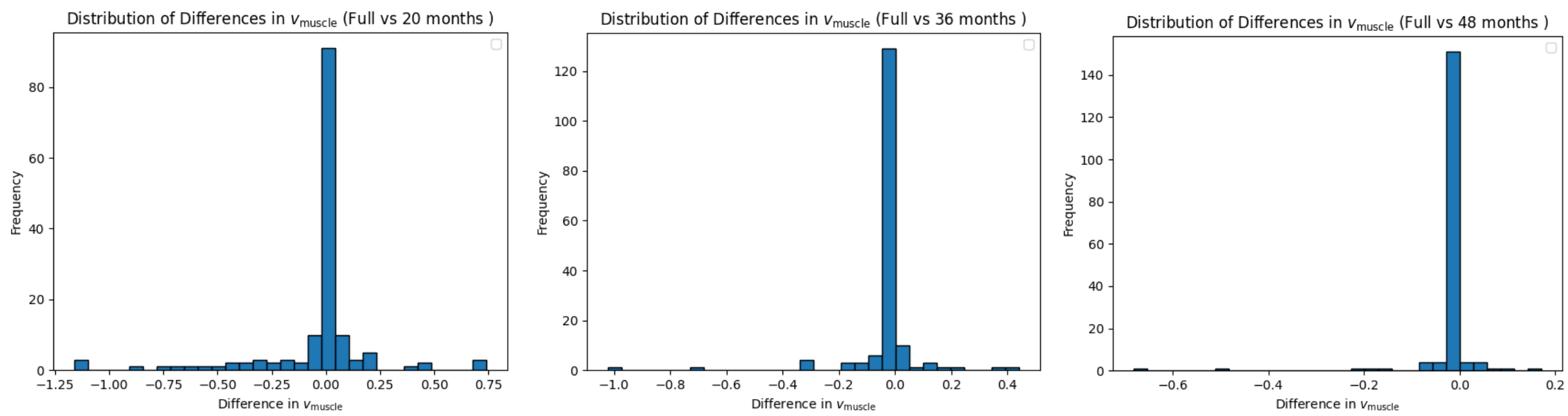

Figure S5. The histogram of velocity difference $v_{full} - v_{cropped}$ between the estimated velocity with all data points and with cropped data points.

Supplementary H Illustration of Cardiac Hypertrophy Cases.

Figure S6 shows two cardiac hypertrophy cases. Figure S6(a) shows the pretreatment scans and Figure S6(b) shows the corresponding last follow-up scans. The last post-treatment scan demonstrates a reduction in left lung volume, likely related to treatment-associated volume loss. This is accompanied by an apparent increase in cardiac size and/or leftward cardiac displacement/rotation within the thorax.

There are several plausible explanations for this finding. First, treatment-related pulmonary injury, fibrosis or pleural disease can reduce pulmonary reserve and increase heart pressure. Second, an increase in LV myocardial volume may reflect treatment-related cardiotoxicity, or a late sequel of pulmonary hypertension. Finally, because the scans were not ECG-gated cardiac acquisitions, some component of the measured change may reflect differences in cardiac phase, motion artifact, or segmentation uncertainty.

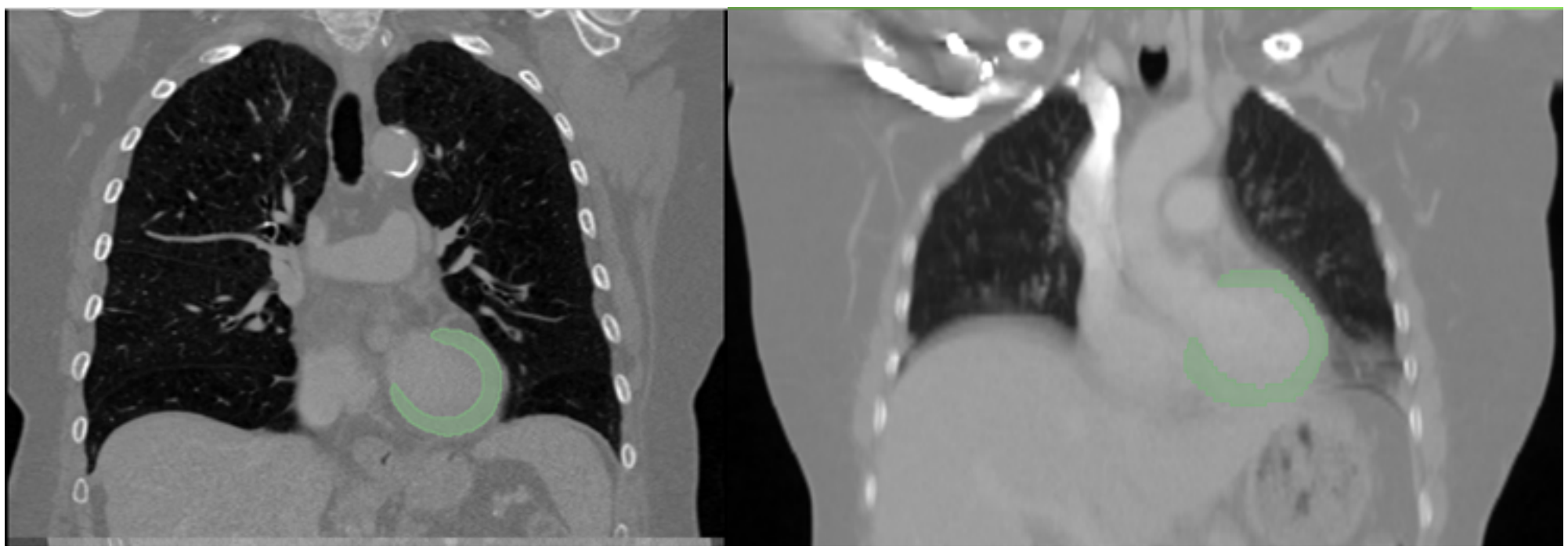

(a) Pre-treatment scans

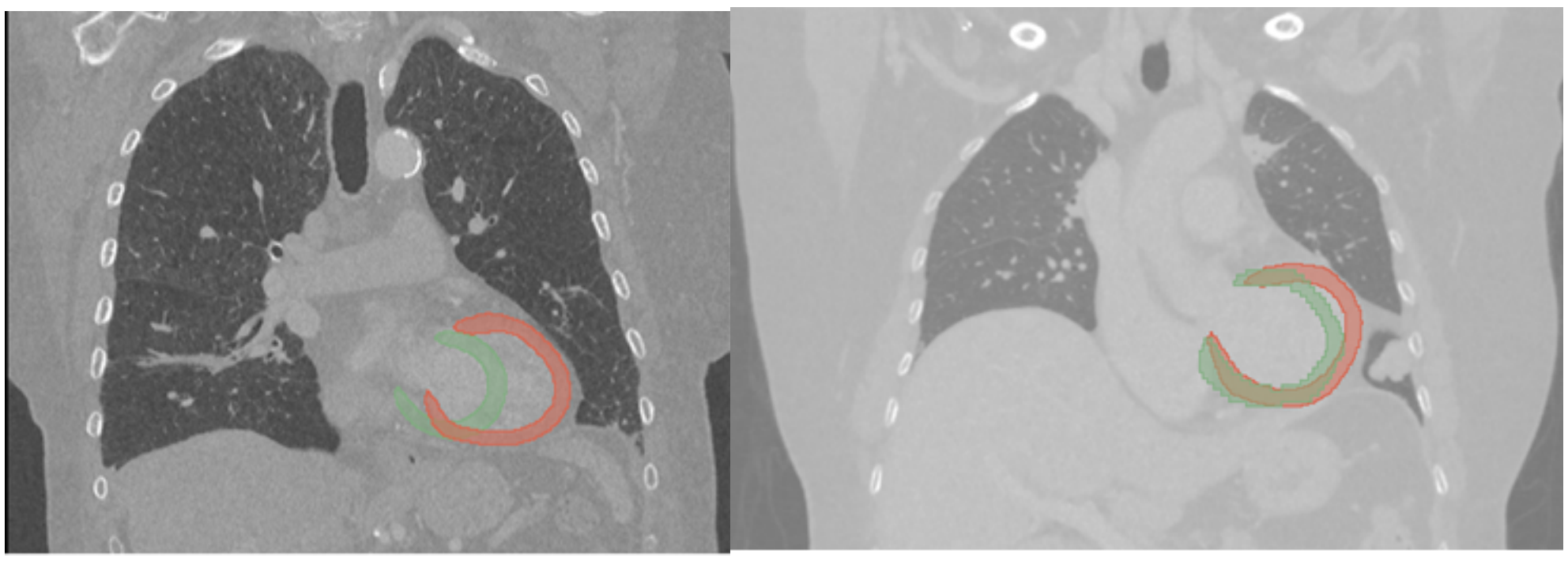

(b) The last follow-up scans

Figure S6. Two cardiac hypertrophy cases. (a) shows the pretreatment scans. (b) shows the corresponding last follow-up scans.
